# Effect of Single-Crystal TiO_2_/Perovskite Band Alignment on the Kinetics of Electron Extraction

**DOI:** 10.1021/acs.jpclett.3c03536

**Published:** 2024-02-15

**Authors:** Xiangtian Chen, Hannu P. Pasanen, Ramsha Khan, Nikolai V. Tkachenko, Csaba Janáky, Gergely Ferenc Samu

**Affiliations:** † Department of Physical Chemistry and Materials Science, Interdisciplinary Excellence Centre, University of Szeged, Aradi Square 1, Szeged H-6720, Hungary; ‡ Photonic Compounds and Nanomaterials, Chemistry and Advanced Material Group, Tampere University, Tampere FI-33720, Finland; § ELI ALPS, ELI-HU Non-Profit Ltd., Wolfgang Sandner street 3., Szeged H-6728, Hungary; ∥ Department of Molecular and Analytical Chemistry, University of Szeged, Dóm square 7-8, Szeged H-6721, Hungary

## Abstract

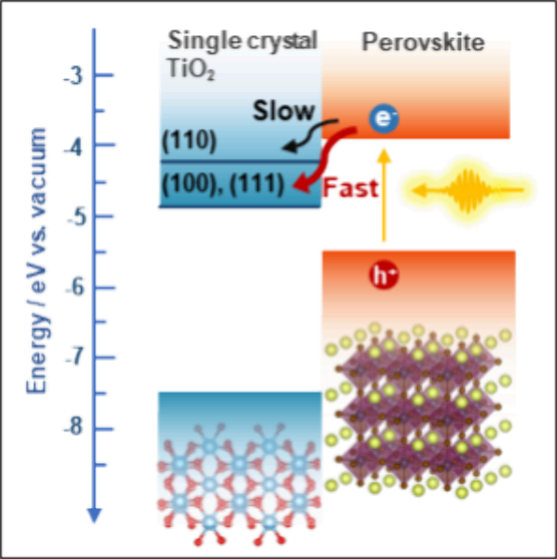

The kinetics of electron extraction at the electron transfer layer/perovskite interface strongly affects the efficiency of a perovskite solar cell. By combining transient absorption and time-resolved photoluminescence spectroscopy, the electron extraction process between FA_0.83_Cs_0.17_Pb(I_0.83_Br_0.17_)_3_ and TiO_2_ single crystals with different orientations of (100), (110), and (111) were probed from subpicosecond to several hundred nanoseconds. It was revealed that the band alignment between the constituents influenced the relative electron extraction process. TiO_2_(100) showed the fastest overall and hot electron transfer, owing to the largest conduction band and Fermi level offset compared to FA_0.83_Cs_0.17_Pb(I_0.83_Br_0.17_)_3_. It was found that an early electron accumulation in these systems can have an influence on the following electron extraction on the several nanosecond time scale. Furthermore, the existence of a potential barrier at the TiO_2_/perovskite interface was also revealed by performing excitation fluence-dependent measurements.

Lead halide perovskites are promising materials in the field of light conversion devices (e.g., solar cells,^1^ photodetectors,^2^ and light-emitting diodes^3^). Among these, perovskite solar cells (PSCs) have received the most scientific and industrial interest. In the past decade, the efficiency of PSCs has surged from 14% to 26%. This feat was achieved through (i) perovskite composition tuning,^4^ (ii) synthesis process engineering,^5^ (iii) developing surface treatment strategies,^6^ and (iv) controlling the microstructure^7^ of the perovskite thin films. These strategies focused on improving the quality of the perovskite material itself. However, attention must be equally given to the charge transfer layers and the formed interfaces^8^ within perovskite-based devices. The charge separation and extraction process occurring at the charge transfer layer/perovskite interfaces also play an important role in determining the overall performance of the PSCs, which cannot be neglected.^9^ The unbalanced electron or hole transfer coupled with large interface defect densities (trap states) can result in unfavorable charge accumulation at these interfaces, prevalent in many state-of-the-art PSC architectures.^10^ Charge accumulation at the interfaces of PSCs has a correlation with severe current–voltage hysteresis^11^ and low open-circuit voltage^12,13^ and can compromise the long-term stability of devices. Therefore, optimizing charge transfer layer/perovskite interfaces to tackle these issues is critical for realizing high-efficiency and stable PSCs.

High-performance PSCs in n-i-p configuration are based on TiO_2_ electron transfer layers (ETLs)^14,15^ because of its wide band gap (minimal parasitic light absorption), suitable conduction band (CB) position, high thermal stability, and low cost.^16^ Nevertheless, the further improvement of PSCs based on TiO_2_ ETL is hindered by its slow electron mobility^17^ and relatively high density of surface trap states (e.g., oxygen vacancies), which can either cause fast carrier recombination or electron accumulation at its interface.^18^ Moreover, PSCs employing TiO_2_ ETLs have a critical instability arising from light-induced desorption of surface-adsorbed oxygen.^19^ Different modification methods were developed to mitigate the previous issues. Doping elements (e.g., Li, Nb, and Mg) can improve electrical conductivity while simultaneously tuning the band offset, enabling more efficient charge extraction.^20^ Other methods, like surface treatment (e.g., TiCl_4_ treatment)^21^ and interface engineering (passivation with different layers such as MgO, Al_2_O_3_, ZrO_2_ and C_60_),^22^ were also demonstrated as effective strategies to suppress recombination at the TiO_2_/perovskite interface. A similarly effective strategy in influencing the performance and stability is crystal-facet engineering of the ETLs in PSCs. This strategy was already demonstrated in photoelectrochemistry^23^ and photocatalysis^24^ energy conversion scenarios. Fundamental understanding of charge carrier dynamics at the specifically oriented TiO_2_/perovskite interfaces is critically important for tailoring the properties (e.g., crystallinity) of the TiO_2_ ETLs.

Electron extraction from perovskite layers to TiO_2_ ETLs is generally considered to occur in the early time scales. The specific electron transfer rate varies from picoseconds^25,26^ to several tens of nanoseconds^27,28^ depending on the structure, surface area, and synthesis process of TiO_2_ ETLs (e.g., ETLs in planar, mesoporous, and nanorods forms^29^ or ETLs annealed in different atmospheres^30^). Furthermore, experimental conditions can also influence the determined electron extraction rate constants. For example, the applied laser excitation intensity has an impact on both charge recombination and charge transfer processes at the TiO_2_/perovskite interface.^31,32^ A potential barrier is shown to form at the TiO_2_/perovskite interface, which leads to charge accumulation, hindering electron transfer.^33^ The kinetics of the charge recombination and extraction processes can also be influenced by the perovskite film quality, such as grain size,^34,35^ quantity of impurity phases,^36,37^ and interface and surface defect densities.^38,39^ In the case of front-side excitation (from the perovskite/air interface), the carrier diffusion process also needs to be taken into consideration before these carriers can be extracted by the ETL.^40−42^ It is challenging to ensure the similarity of these parameters and present a meaningful comparison of charge carrier dynamics (and carrier extraction) in perovskites and at related interfaces. When using single-crystal ETLs, at least variation arising from the structure and surface area of the ETLs can be mitigated. Orientation-dependent carrier extraction from MAPbI_3_ has been demonstrated using single-crystal TiO_2_ surfaces.^28^ Electron transfer from MAPbI_3_ to rutile TiO_2_ is more efficient for the (100) and (110) facets than for the (001) facets. However, variation in perovskite layer quality (density of trap states, grain size differences), can also lead to different carrier recombination kinetics.

In this study we aimed to characterize the kinetics of the electron extraction process from FA_0.83_Cs_0.17_Pb(I_0.83_Br_0.17_)_3_ perovskite thin films to rutile TiO_2_ single crystals, where three different orientations of TiO_2_ single crystals were studied. This type of perovskite was chosen because of its prominence in efficient and stable PSCs.^43,44^ The perovskite layers were carefully optimized to have identical quality (e.g., crystallinity, phase purity, and average grain size) on all three different rutile TiO_2_ surfaces possessing an orientation of (100), (110), and (111). To link the variation in the electron extraction properties of these interfaces to the electronic properties of the TiO_2_ single crystals we also determined the exact band positions by ultraviolet photoelectron spectroscopy (UPS) and UV–vis spectroscopy. Furthermore, the Fermi level of the substrates were also obtained by Kelvin probe measurements. By combining transient absorption spectroscopy (TAS) and time-resolved photoluminescence spectroscopy (TRPL), we tracked the kinetics of the electron extraction process at the TiO_2_/perovskite interface from 0.1 ps to 300 ns. We observed the fastest electron extraction in the case of the TiO_2_(100) facet. We found that the rate of electron extraction can be directly correlated with the conduction band offset and Fermi level difference at the TiO_2_/perovskite interface. These results can give guidance for the better design of high-performance TiO_2_ ETLs for PSCs.

To separate the electron extraction process with TAS and TRPL measurements, control over the perovskite layer properties (e.g., film thickness, crystallinity, and grain size) had to be ensured. As the initial step, we performed detailed characterization of the rutile TiO_2_ single crystals with specific orientations of (100), (110), and (111) used in this study. The orientations of the single-crystal substrates were confirmed by XRD measurements^45,46^ (Figure S1). AFM measurements (Figure S2) were performed to reveal that the single-crystal substrates had a surface roughness of 0.1 nm for TiO_2_(100), 0.3 nm for TiO_2_(110), and 0.3 nm for TiO_2_(111), respectively. As a next step, FA_0.83_Cs_0.17_Pb(I_0.83_Br_0.17_)_3_ (termed perovskite in the text) was spin-coated on top of these single-crystal TiO_2_ substrates. Additional samples on glass substrates were also prepared to serve as a reference in the charge transfer studies (in this case no charge extraction occurs). As the structural and optical properties of the perovskite films are sensitive to the used substrates (e.g., mesoporous substrate versus flat substrate^47,48^), ultraflat quartz-coated glass was used. The deposition procedure of the perovskite layers was carefully optimized to obtain the same absorbance throughout the whole UV–vis range on the different substrates (Figure S3). This process becomes increasingly difficult when targeting the deposition of a thin film (<100 nm thick). The detailed preparation protocol of the perovskite thin films can be found in the Supporting Information. Special attention was devoted to select the optimal annealing conditions (number of steps, temperature of each step, and duration), while monitoring the phase composition and morphology of the perovskite films. In a similar fashion the solution composition (DMF/DMSO ratio, antisolvent volume, nature, and treatment time) was also scrutinized to obtain phase-pure perovskite films. The XRD patterns (Figure S4) of the perovskite layers on the single-crystal TiO_2_ substrates revealed the formation of the desired perovskite phase in all cases. Characteristic diffraction peaks of FA_0.83_Cs_0.17_Pb(I_0.85_Br_0.17_)_3_ were identified,^49^ with a slight shift compared to pure FAPbI_3_ because of the incorporation of Cs and Br into the lattice, in good agreement with previous literature examples.^44^ Apart from the reflections from the TiO_2_ substrates, no impurity phases were detected. To characterize the thickness of the perovskite layers, ellipsometry measurements were performed (Table S1). Except for the glass/perovskite sample (110 nm thickness), identical thicknesses were determined for the films deposited on the TiO_2_ single-crystal substrates (90 nm). The surface roughness was around 2 nm in all cases (as deduced from ellipsometry). These results suggest that all perovskite layers have similar thicknesses and smooth surfaces. To assess the grain size of the prepared perovskite thin films we recorded top-down SEM images (Figure S5). The SEM images and grain size distributions of the prepared perovskite layers on different substrates also showed identical grain sizes (60 nm). These results demonstrate that all vital parameters regarding the morphology of samples that can influence charge carrier dynamics in these systems were kept under control.

To characterize the electronic properties of TiO_2_ single crystals with different orientations, ultraviolet photoelectron spectroscopy (UPS) (Figure S6 and Table S2) measurements were carried out to determine their valence band (VB) positions. Comparing the VB position of the TiO_2_ single crystals (Figure 1A) identical energy levels were determined for the (100) and (111) orientations (at −7.8 eV), while a more positive energy level was determined (at −7.2 eV) for the (110) orientation. To obtain the CB position of the TiO_2_ substrates the bandgap was determined from UV–vis spectra of the substrates coupled with Tauc-analysis (Figure S7). The direct bandgap of the rutile single-crystal TiO_2_ was determined to be 3.0 eV in all cases. This preserves the same offset between the CB energy levels as the ones determined for the VB of these substrates. (Figure 1A). As a next step, we turned our attention to the assemblies where the perovskite layer was deposited on the different substrates. The VB position of perovskite layers on different substrates were characterized by ambient-pressure photoemission spectroscopy (APS). In all cases, a VB position of −5.5 eV (see Figure S8) was determined, which is in good agreement with the literature value of −5.7 eV obtained by UPS.^50^ We determined the CB of the perovskite layers in a similar fashion as demonstrated for the pristine TiO_2_ substrates. Tauc analysis of the UV–vis spectra (Figure S3) of the perovskite layers on the different substrates (Figure S9) revealed a direct bandgap of 1.7 eV. Therefore, the CB position of perovskite layers on all substrates is calculated to be −3.8 eV (Figure 1A).

**Figure 1:**
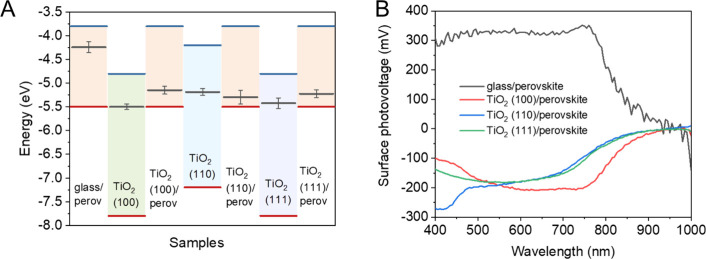
(A) Band positions and Fermi level alignment of glass/perovskite sample and single-crystal TiO_2_/perovskite samples. Error bars are obtained from measurements performed on three different samples. (B) Surface photovoltage spectroscopy of glass/perovskite sample and single-crystal TiO_2_/perovskite samples.

The CB position differences at TiO_2_(100)/perovskite and TiO_2_(111)/perovskite interfaces are larger (Δ*E* = 1.0) than in the case of the TiO_2_(110)/perovskite interface (Δ*E* = 0.4). This implies a larger thermodynamic driving force for the electron extraction process in the former cases. To determine the Fermi-level of the samples, contact potential difference (CPD) measurements were carried out. The corresponding Fermi levels of single-crystal TiO_2_ samples were −5.5 ± 0.06, – 5.2 ± 0.07, and −5.4 ± 0.1 eV for the (100), (110), and (111) facets, respectively (Figure 1A). The position of the Fermi level is in good agreement with the n-type nature of these materials. The perovskite layer on glass substrate shows a Fermi level of −4.2 ± 0.1 eV. When the perovskite is deposited on the surface of the TiO_2_ single crystals, the measured Fermi level is pinned to −5.2 ± 0.1 eV. This is not surprising, as the perovskite layer thickness in this study was around 100 nm, and the depletion width of MAPbI_3_ on TiO_2_ can reach ∼300 nm.^51^ This results in the depletion region spanning through the whole layer, leading to the shift of the surface Fermi level.^52^

To monitor the effect of the electron extraction process on the surface photopotential of the samples, surface photovoltage spectroscopy (SPS) measurements were carried out. When the perovskite layer is illuminated by light with energy exceeding the optical bandgap, photoexcited charge carriers are generated. This is followed by the redistribution of the photogenerated carriers in the perovskite layer, which causes the change in the Fermi level of the surface. SPS spectra of the perovskite layers on different substrates are shown in Figure 1B. On glass substrates, the perovskite layer shows a positive photovoltage, indicating an increased number of electrons at the surface. These electrons are either located in the space charge region or trapped at surface states.^53,54^ In contrast, when the perovskite layer is interfaced with TiO_2_ substrates, it shows a negative photovoltage, meaning that electrons are effectively extracted to TiO_2_ substrates, while holes are left on the surface. This further confirms that rutile TiO_2_ single crystals can be used as electron extraction layers in perovskite-based light-harvesting devices. From the rise of the photovoltage in the case of all substrates, a lower bandgap (1.44 eV) can be deduced than from optical measurements, which signals the presence of surface trap states.

To understand the kinetics of the charge carrier extraction process after excitation, both steady-state PL and TRPL measurements were carried out. The position of the PL maximum of the samples was at 750 nm, which is in good agreement with the typical value for this perovskite composition. Compared to glass/perovskite, the steady-state PL intensity was quenched to 29.4%, 44.3%, and 33.1% when the perovskite was interfaced with TiO_2_ (100), (110), and (111), respectively (Figure 2A and Table S3). The observed PL intensity is proportional to the excited charge carrier population in the perovskite layer (which can undergo radiative recombination). Both charge extraction and surface defect-assisted charge recombination can partially deplete the excited charge carrier population, leading to quenching of the PL signal. As the perovskite layer properties were carefully controlled, we assign the decrease of the PL signal to mainly electron transfer to the TiO_2_ substrates (as all samples have proper band alignment for electron extraction). The magnitude of the PL quenching is in good correlation with the previously determined CB offset between the TiO_2_/perovskite. A larger quenching of the PL signal was observed when larger offset was determined between the TiO_2_ and the perovskite. TRPL decay curves can further support this notion (Figure 2B). Although TRPL decay curves monitor the radiative electron–hole recombination process, other competitive nonradiative recombination and charge extraction processes can influence the observed decay pattern. The PL decay curves were fitted with a triexponential function, and the extracted parameters are shown in Table S4. The initial assessment of the decay curves was performed based on the comparison of the average lifetimes, which is a model-independent value. Generally, a shorter average lifetime was determined for the perovskite deposited on TiO_2_ substrates compared to the glass substrate (τ_avg_ = 24.0 ns), which is also apparent from the PL decay curves (Figure 2B). Also, the shortening of the average lifetime (Table S4) and the relative PL yield (Table S3) in TiO_2_/perovskite samples are in good agreement. Considering the perovskite layer quality is the same on these substrates, the accelerated PL decay on TiO_2_ substrates indicates the contribution of electron extraction to the overall PL decay. This process influences the PL decay of the perovskite layer deposited on TiO_2_(100) the most (τ_avg_ = 8.8 ns), which is in good correlation with the large CB difference at this specific interface. TiO_2_(110) was found to influence the PL decay process the least (τ_avg_ = 16.7 ns), where the CB difference was the smallest. In the triexponential fitting the longest lifetime component can be attributed to direct band-to-band recombination (∼100 ns),^29,55,56^ the middle component to trap-state-assisted recombination (∼30 ns), while the shortest one (<10 ns) to a mixture of electron extraction and trap-state-assisted recombination. Note that the order of the shortest lifetime components does not follow the same trend as the order found for the average lifetimes (Table S4). However, the relative contribution of the shortest lifetime component to the overall decay trace seems to follow the trend dictated by the CB offsets between the TiO_2_/perovskite. To assess how the determined PL quenching and lifetime values compare to TiO_2_-based ETLs used in perovskite solar cells, we prepared two different TiO_2_-coated substrates (blocking (bl-TiO_2_) and mesoporous (mp-TiO_2_)). From the steady-state PL spectra (Figure S10A), it is apparent that the quenching efficiency of the bl-TiO_2_ layer is small compared to the single-crystal TiO_2_ substrates. In stark contrast, when utilizing a mp-TiO_2_ layer, similar quenching efficiency can be obtained as the TiO_2_(111) and TiO_2_(100) substrates despite its larger contact area with the perovskite material. This trend is reflected in the PL decay of the samples as well (Figure S10B). Interestingly, the two single-crystal orientations (111) and (100) have faster electron transfer than the prepared mp-TiO_2_ layer. This points toward the fact that apart from having a large contact area between the light absorber and the ETL, attention must be paid to the CB offset between these constituents. To monitor and assess the contribution of electron extraction on this short time scale, TAS measurements were performed.

**Figure 2:**
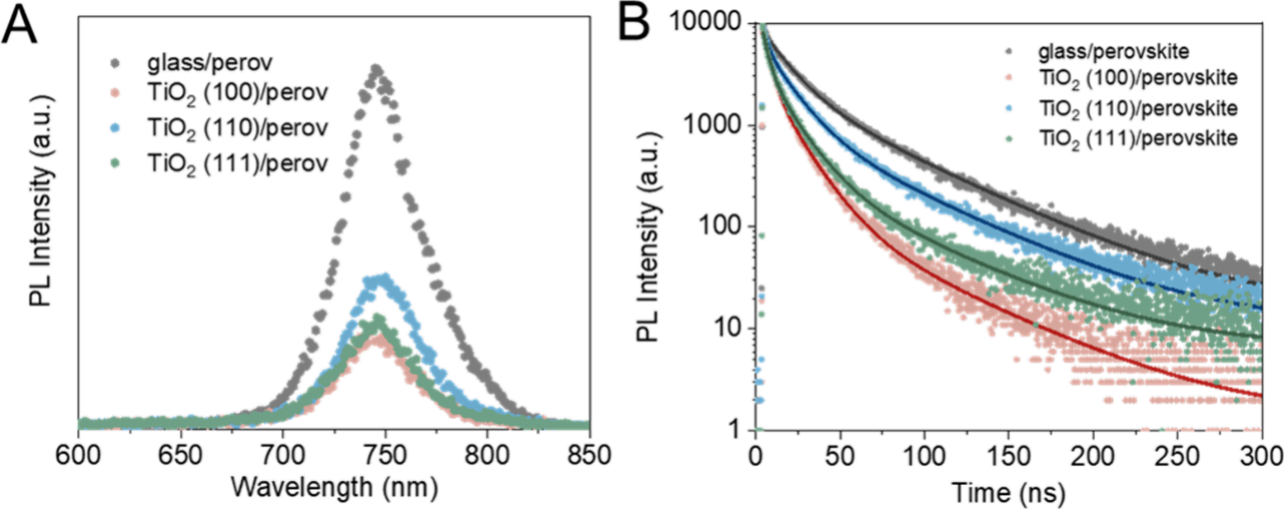
Steady-state PL (A) and TRPL decay curves recorded at 750 nm (B) of perovskite layers deposited on different substrates. Excitation wavelength: 467 nm. Excitation fluence: 29 nJ/cm^2^.

TAS measurements are capable of probing processes at a faster (sub ns) time scale. Global analysis was carried out to separate the individual processes contributing to the overall decay of the excited state. In this manner individual spectral features can be assigned to the components with different lifetimes participating in the multiexponential decay of the TAS signal. Figure 3 shows the decay-associated spectra (DAS) of glass/perovskite samples as examples. The DAS of the other samples can be found in Figure S11A–C. Global analysis revealed three components that contribute to the overall TAS signal. In the case of glass/perovskite samples, the shortest process (0.2 ps) arises from hot carrier cooling which generally finishes within 1 ps. The magnitude of the middle component (53 ps) is relatively small. The long component (6.1 ns) is the characteristic ground-state bleach (GSB) peak of the perovskite, related to the formation of free carriers after excitation. This negative peak decreases over time as carriers recombine, are trapped on trap states, or are extracted by the TiO_2_ layer (Figure 3B and Figure S11D–F). By tracking the decay of the GSB and comparing the result between glass and TiO_2_ substrates, electron transfer properties can be revealed.

**Figure 3:**
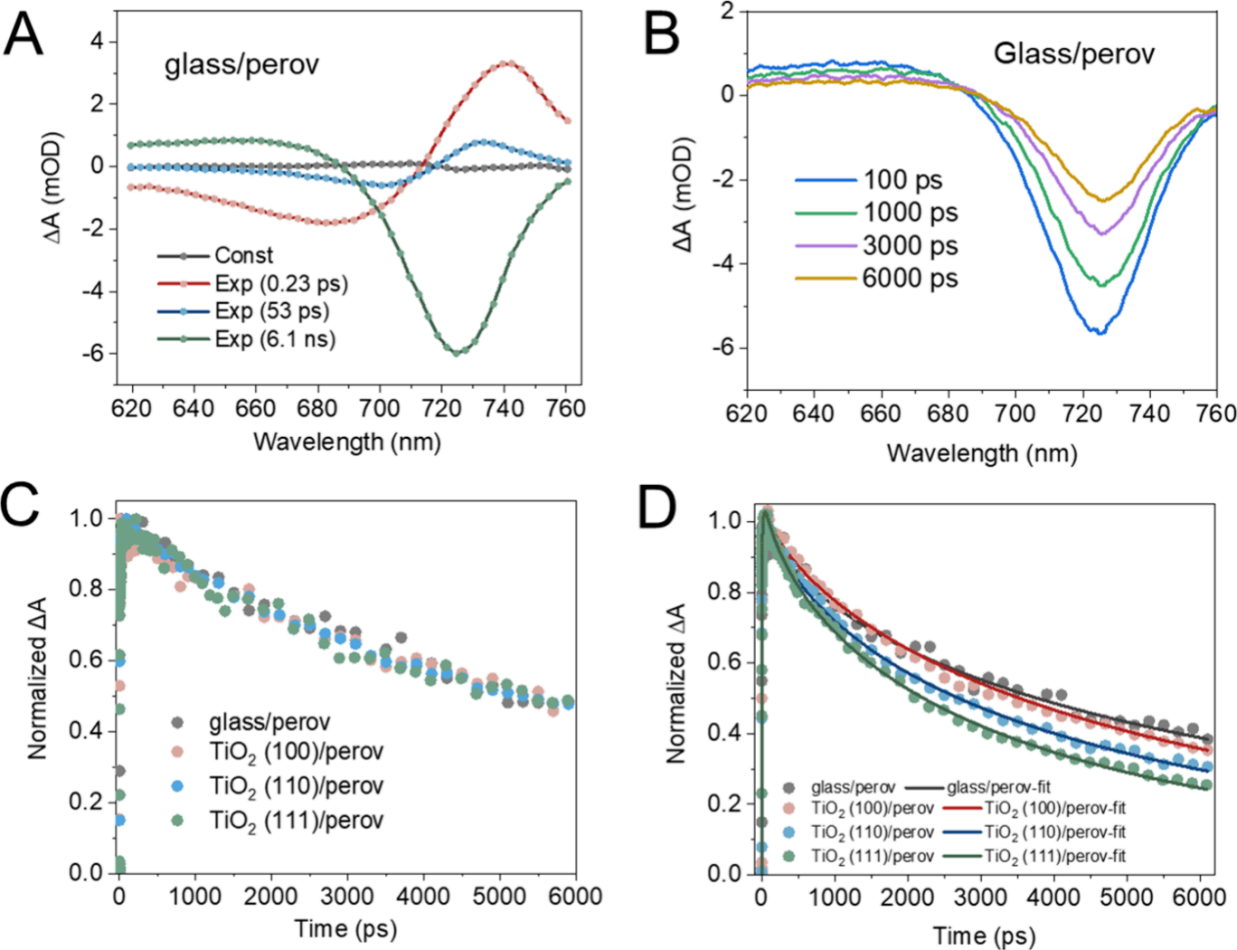
Decay-associated spectra (DAS) (A) and transient absorption spectra (TAS) at various time delays (B) of glass/perovskite sample. Transient absorption (TA) decay traces at 725 nm for perovskite on different substrates excited by a 600 nm wavelength laser at the fluence of 2.8 μJ/cm^2^ (C) and 5.7 μJ/cm^2^ (D).

To assess the effect of initial carrier concentration on the electron extraction process we varied the excitation fluence during the TAS measurements. Interestingly, at low excitation fluences (0.7 μJ/cm^2^ and 2.8 μJ/cm^2^), no change in the decay curves for the relaxation of the GSB signal at 725 nm was observed (Figure 3C and Figure S12) among the TiO_2_/perovskite samples. Furthermore, no significant difference was found when compared to a perovskite layer deposited on a glass substrate. This signals that under these low fluences (low initial carrier concentration) no electron extraction occurs in these systems on the nanosecond time scale. When the excitation fluence was increased to 5.7 μJ/cm^2^, the decay curves start to deviate (Figure 3D) when a TiO_2_ ETL was used. The accelerated decay in the case of the TiO_2_/perovskite samples can be related to the additional electron-transfer process. The possible reason for this excitation fluence-dependent electron extraction behavior is the presence of an electric barrier between TiO_2_ and the perovskite layer at the close vicinity of the interface.^33,57^ Electron accumulation at the CB of perovskite is required to overcome the barrier and initiate the electron transfer process at these time scales. Note that in the TRPL measurement even lower (29 nJ/cm^2^) excitation fluence was used; however, the electron extraction was still observable. This discrepancy can be rationalized by the difference in the excitation frequency between the two techniques, where 1 MHz is used in TRPL, compared to the 1 kHz used for TAS. Perovskites were shown to possess trap states with slow detrapping speed (μs time scale).^58^ This slow detrapping causes the photodoping of the samples, which influences the carrier dynamics of these systems. At higher repetition rate, this results in a similar charge buildup at the interface that allows the electrons to overcome the electric barrier. In this situation the trap states remain populated when the next excitation pulse arrives, effectively increasing the charge carrier concentration in the perovskite films.

In the cases where difference in the TAS decay curves could be observed, the decay traces were fitted by a stretched exponential function. The extracted parameters are listed in Table S5. The results show that the stretching parameters (β) are essentially the same for all four samples. This β value tends to represent the dispersion of electron extraction rate at the interface with perovskite layers.^59,60^ The lifetime (τ) follows the trend: τ (glass/perovskite, 6.1 ns) > τ (TiO_2_(100)/perovskite, 5.3 ns) > τ (TiO_2_(110)/perovskite, 4.1 ns) > τ (TiO_2_(111)/perovskite, 3.1 ns). This indicates that TiO_2_(111) has the fastest, while TiO_2_ (100) has the slowest electron extraction speed in the nanosecond time range. The slow electron extraction kinetic of the TiO_2_(100) facet contradicts the trend of the average lifetimes observed with TRPL (in the ∼300 ns time scale). This can be rationalized when even earlier time scales are scrutinized, where the behavior of hot carriers can be followed. The carrier distribution at the interface on the earliest time scales can have an impact on the following electron extraction process.

During above bandgap excitation, hot carriers are generated, which will thermalize to the CB of the semiconductor. The hot electron temperature (*T*_c_) can be obtained by fitting the high-energy tail of the band edge region of the GB signal in the TA spectra^61,62^ (Figures 4A,B, S13, and S14). Details of the fitting procedure can be found in Supporting Information. Figure 4C shows that under low excitation fluence (2.8 μJ/cm^2^) the cooling kinetics of hot carriers in all samples are similar. When the excitation fluence is increased to 5.7 μJ/cm^2^ (see Figure 4D), slower cooling of hot carriers in glass/perovskite samples is observed (electron pileup at higher lying energy regions of the CB). However, this seems to be not present in the case of TiO_2_/perovskite samples. The faster cooling of hot carriers in TiO_2_/perovskite samples can be an indication of hot electron extraction at the near vicinity of the TiO_2_. Among all three single-crystal TiO_2_ facets, TiO_2_(100) shows the fastest hot carrier extraction. This can result in a relatively high concentration of electrons in the CB of TiO_2_(100) after 1 ps. The presence of electrons in the CB of TiO_2_ can bottleneck longer time scale electron extraction events, until these electrons in the CB of TiO_2_ either diffuse away from the interface or participate in back electron recombination events.^63,64^ This explains why we observed the slowest electron extraction in the case of the TiO_2_(100) facet in TAS (in the time range of 1–6 ns). In longer time scales (TRPL results (in the time range of ∼300 ns)), when the electrons can be removed from the CB of TiO_2_, efficient band edge electron extraction can be achieved by the TiO_2_(100) facet.

**Figure 4:**
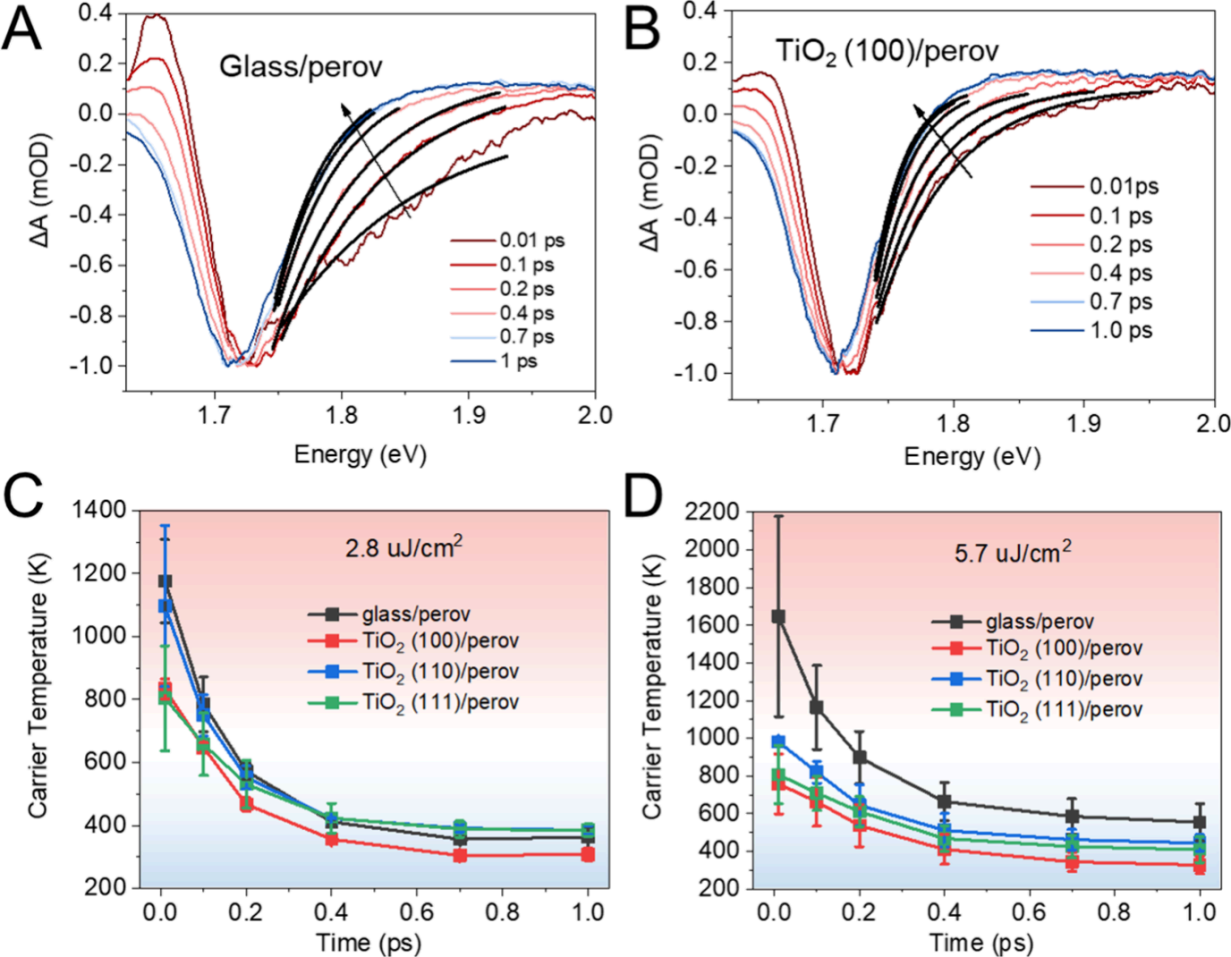
Transient absorption (TA) spectra of glass/perovskite (A) and TiO_2_(100)/perovskite sample (B) at various delay times within 1 ps and hot electron cooling as a function of time at excitation fluence of 2.8 μJ/cm^2^ (C) and 5.7 μJ/cm^2^ (D) with 600 nm excitation.

The influence of the excitation fluence on hot and band edge electron extraction are summarized in Scheme 1. When high excitation fluence is used, the hot electron cooling process slows down, allowing hot electron extraction by the ETL. In a similar fashion, electron accumulation is needed to conquer the electric potential barrier at the TiO_2_/perovskite interface to initiate band edge electron transfer. We would like to highlight the importance of fluence variation in the investigation of charge transfer kinetic studies at the interface of perovskite and its charge transfer layers, which is usually neglected.

**Scheme 1:**
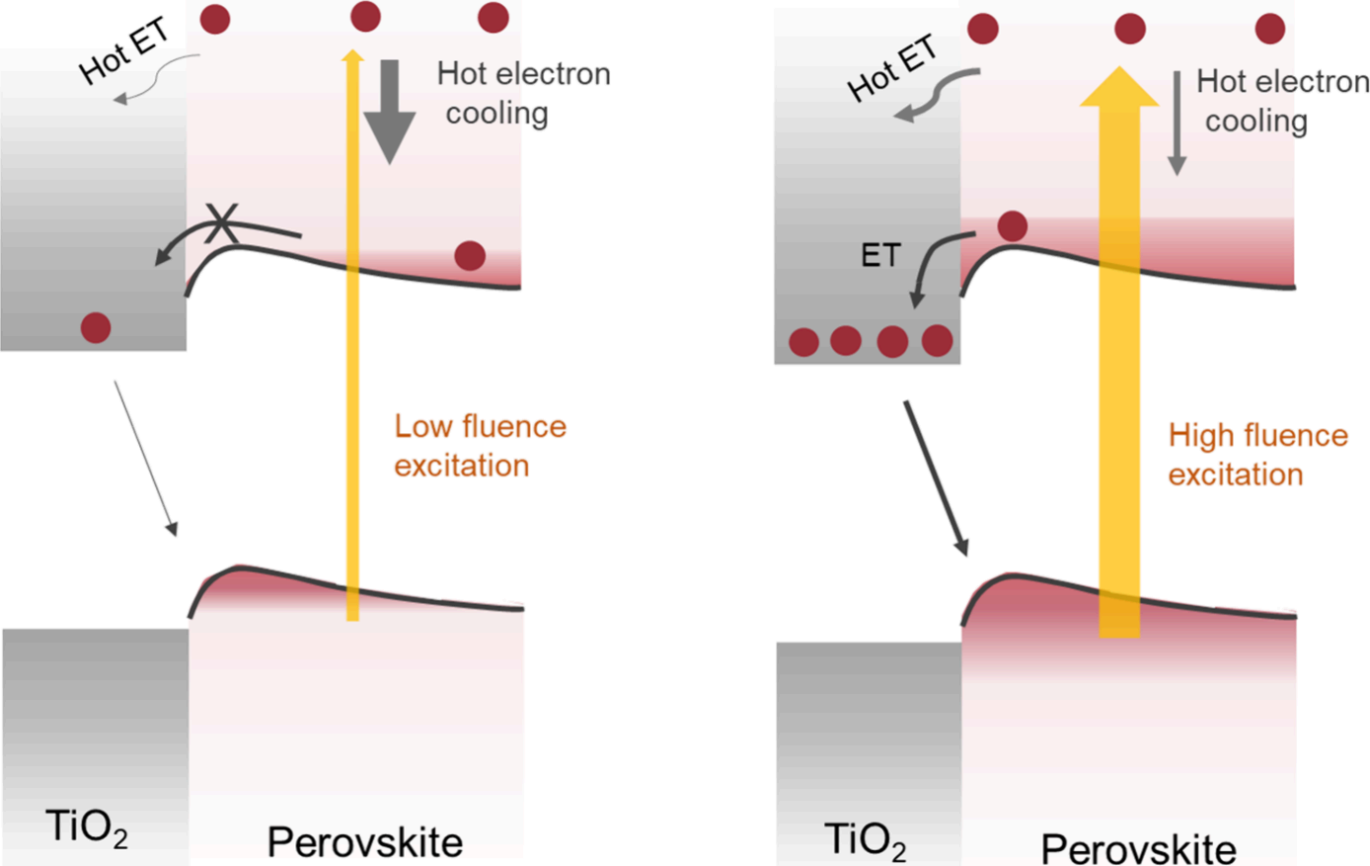
Mechanistic Insights on Electron Transfer at the TiO_2_/Perovskite Interface and How This Process Can Be Influenced by an Electric Barrier at the Interface and Excitation Fluence Variation

In summary, we prepared FA_0.83_Cs_0.17_Pb(I_0.83_Br_0.17_)_3_ perovskite layers with the same quality (phase purity, thickness, and grain size) on glass and rutile TiO_2_ single crystals with three different orientations. We experimentally determined the band diagram of the assemblies, where the largest CB and Fermi level difference (Δ*E* = 1.0) was found for the TiO_2_(100)/perovskite interface. This energy offset ensured fast and efficient electron extraction as shown by the magnitude of PL quenching (29.4%) and the acceleration of the TRPL signal (τ_avg_ = 8.8 ns). This shows that the determined band positions are suitable to rationalize the kinetics of electron extraction processes on the longer time scale in these model systems. However, on the intermediate time scales (<ns) deviation from the thermodynamic picture can be observed, which can be attributed to the differences in hot carrier extraction (ps) in these systems. This highlights the importance of investigating charge transfer processes on wider time scales. Fluence-dependent measurements revealed the presence of an electric barrier at the interface in these systems; as electron transfer was only observed when sufficient electron accumulation at the CB was achieved. Overall, the fast overall electron transfer makes ETLs based on rutile TiO_2_(100) facets attractive candidates for perovskite solar cells. Furthermore, by ensuring transport of accumulated carriers from the TiO_2_(100) CB at the early time scales, contribution from hot carriers could be better utilized. However, when translating electron transfer rates in different ETL/perovskite compositions to solar cell efficiencies, charge transport processes must also be considered. Altering the charge transfer layers’ (CTLs’) chemical composition (e.g., doping, surface passivation, alloying) or using novel materials inherently modifies multiple properties of the CTLs not just the band edge energies. Unintentional alterations to the conductivity, trap states’ properties (number, energy position), or the subsequently deposited perovskite layer properties all play a role in influencing solar cell efficiencies. This points toward the necessity of more fundamental studies, where at least some of these effects can be disentangled from the complex picture.

## Supporting Information Available

Experimental details on the preparation of the perovskite thin films and details of the characterization procedures; XRD patterns, AFM images, UPS spectra of TiO_2_ single-crystal surfaces, SEM images and grain size distribution graphs of the deposited perovskite layers, optical characterization of the samples (UV–vis spectra and Tauc-plots); additional figures related to the characterization of the ultrafast processes such as transient absorption spectra and decay-associated spectra of TiO_2_(110)/perovskite and TiO_2_(111)/perovskite samples and decay traces regarding fluence-dependent measurements; detailed tables containing the obtained fitting parameters for the TRPL and TAS traces; hot carrier temperature determination procedure (PDF)
