# Supplementary material for: Effect of Single-Crystal TiO2/Perovskite Band Alignment on the Kinetics of Electron Extraction
Source: J Phys Chem Lett. 2024 Feb 15;15(7):2057–65. doi: 10.1021/acs.jpclett.3c03536 (PMC10895670; doi:10.1021/acs.jpclett.3c03536)
Supplement: Supplementary file 1 — jz3c03536_si_001.pdf [file jz3c03536_si_001.pdf]

Supporting Information

The Effect of Single Crystal TiO_2/Perovskite Band Alignment on the Kinetics of Electron Extraction

Xiangtian Chen[1], Hannu P. Pasanen[2], Ramsha Khan[2], Nikolai V. Tkachenko[2], Csaba Janáky[1,3*], Gergely Ferenc Samu[3,4*]

[1]Department of Physical Chemistry and Materials Science, Interdisciplinary Excellence Centre, University of Szeged, Aradi Square 1, Szeged, H-6720, Hungary

[2]Photonic Compounds and Nanomaterials, Chemistry and Advanced Material Group, Tampere University, Tampere, FI-33720, Finland

[3]ELI ALPS, ELI-HU Non-Profit Ltd., Wolfgang Sandner street 3., Szeged, H-6728, Hungary

[4]Department of Molecular and Analytical Chemistry, University of Szeged, Dóm square 7-8, Szeged, H-6721, Hungary,

*Address correspondence to these authors: janaky@chem.u-szeged.hu, samugf@chem.u-szeged.hu

1. Experimental Section

1.1. Used Chemicals

Rutile single crystal TiO_2 substrates with (100), (110), and (111) specific orientations were purchased from Mateck (1 cm x 1 cm x 1 mm). The glass substrates are ultra-flat quartz coated glasses purchased from Ossila. For the preparation of mixed perovskite layers, lead iodide (PbI_2, Alfa Aesar, ultra dry, 99.999% trace metals basis), lead bromide ($PbBr_2$, Alfa Aesar, Puratronic®, 99.998% trace metals basis), formamidinium iodide (FAI, Greatcellsolar), cesium iodide (CsI, Thermo Scientific, 99.9% trace metals basis), dimethyl sulfoxide (DMSO, Sigma-Aldrich, anhydrous, >99.9%), n,n-dimethyl-formamide (DMF, Sigma-Aldrich, anhydrous, 99.8%), and chlorobenzene (C_6H_5Cl, Sigma-Aldrich, anhydrous, 99.8%) were used without further purification. For the preparation of blocking TiO_2 and mesoporous TiO_2, titanium diisopropoxide bis(acetylacetonate) (Sigma-Aldrich, 75 wt. % in isopropanol), 1-butanol (Sigma-Aldrich, 99.8%), TiO_2 paste (Sigma-Aldrich, 19.0 wt. %), and $TiCl_4$ (Alfa Aesar, 99.6%) were used.

1.2. Layer Preparation

The single crystal TiO_2 and glass substrates were always cleaned by the following procedure before use: thorough cleaning with surfactant, sonication in DI water, acetone and isopropanol, 15 min each. Then the substrates are subjected to UV ozone plasma cleaning for 15 min. The cleaned substrates are immediately transferred into N_2 filled glove box for film preparation.

TiO_2 blocking layer is spin coated on FTO substrate from a 0.15 M titanium diisopropoxide bis(acetylacetonate) solution in 1-butanol. A 3 step dynamic coating procedure was used: (1) at 700 rpm for 8 s, (2) at 1000 rpm for 10 s and (3) 2000 rpm for 40 s (acceleration 1000 rpm). 0.5 cm^{-3} of bl-TiO_2 solution was used. The layer was then subjected to heat treatment for 5 minutes at T=125°C. In the case of mesoporous TiO_2 substrates, a layer of mesoporous TiO_2 was spin coated on top of FTO/TiO_2

blocking layer from an ethanol based TiO_2 paste suspension with c=0.12 g cm^{-3}. 1 step static coating procedure at 2000 rpm for 20 s (acceleration 1000 rpm) was used. All procedures mentioned above were carried out in a N_2 filled glove box. The spin coated mesoporous TiO_2 layer is then calcined for 1 hour at T=550 °C in air. The FTO/TiO_2 blocking layer/mesoporous TiO_2 samples were then subjected to a $TiCl_4$ treatment step. For this, aqueous solution of 20 mM $TiCl_4$ was prepared, and the samples were immersed into it at T=90 °C for 15 minutes. This step was followed by a final calcination step for 30 minutes in a preheated muffle furnace at T=500 °C in air.

The mixed perovskite ($FA_{0.83}Cs_{0.17}Pb(I_{0.85}Br_{0.15})_3$) layers were prepared by a one-step spin coating method. The precursor solutions were formed by adding 0.1713 g FAI, 0.0530 g CsI, 0.4121g PbI_2, and 0.1123 g $PbBr_2$ in DMF and DMSO mixture solution (volume ratio 7:3) to make a 1 mL 1.0 M solution. Prior to spin coating, the solution was left to stir for at least 1 h at 70 °C on a hot plate and was filtered with a 0.2 µm PTFE filter before use. This solution was then diluted into the desired concentration (0.25 M for perovskite layer on single crystal TiO_2 and 0.3 M for perovskite layer on glass substrate) to prepare thin films (around 100 nm), which allows transient absorption spectroscopic characterization. 16 µl of perovskite precursor solution was used for spin coating. Two-step spin coating procedure was used: (1) 1000 rpm for 10 s (1000 rpm acceleration), (2) 4000 rpm for 30 s (1200 rpm acceleration). At the last 10 s of the second step, 100 µl of chlorobenzene (Sigma-Aldrich, anhydrous, 99.8%) was dispensed on the spinning substrate. After spin-coating, the thin films were subjected to annealing on a 80 °C hot plate for 2 min to form the desired phase, while avoiding the formation of PbI_2. All processes were carried out in a nitrogen filled glove box to ensure inert conditions (<0.1 ppm H_2O, <10 ppm O_2). The prepared layers were stored under these conditions and were used immediately after preparation.

1.3. Material Characterization

X-ray diffraction (XRD) measurements were carried out with a Bruker D8 Advance instrument with a Cu Kα (λ = 1.5418 Å) X-ray source in the 10-80° 2 theta range for bare single crystal TiO_2 and 10-46° 2 theta range for perovskite layers with a scan speed

of 1° min^{-1}. Top-down scanning electron microscopic (SEM) images were captured using a FEI Helios NanoLab DualBeam instrument. Atomic force microscopy (AFM) was measured by NT-MDT Solver AFM microscope. Contact potential difference (CPD) measurements were carried out with a KP Technology APS04 instrument, with a vibrating gold alloy-coated tip. The used gold tip has a Fermi level of −4.78 eV, the Fermi level of single crystal TiO_2 and perovskite layers on different substrates are calculated by equation: E_f (sample)=E_f (gold tip) + CPD[1]. Surface photovoltage spectroscopy (SPS) measurements were carried out with the same instrument, while illuminating the sample surface, with light. By subtracting CPD value measured in the dark from the CPD under illumination surface photovoltage was determined. The wavelength of illumination was scanned from 1000 to 400 nm. Ambient-pressure photoemission spectroscopy (APS) was carried out with a stationary Kelvin-probe tip, illuminated with variable energy UV light source. Ultraviolet photoelectron spectroscopy (UPS) was performed with a He (I) excitation (21.22 eV) source. There was 10 V of external bias applied to the samples to accelerate secondary electrons to the analyzer.

1.4. Optical Characterization

Steady state UV–vis absorption spectra of the prepared thin films were recorded with an Agilent 8453 UV–visible diode array spectrophotometer. Steady state and time resolved photoluminescence (TRPL) measurements were carried out using a Horiba DeltaPro with a 467 nm laser source. The applied laser pulse frequency was 500 kHz. Roughly 90% of the laser output was used to pump an optical parametric amplifier (Topas C, Light Conversion Ltd.) used to produce excitation pulses. Transient absorption spectroscopy was performed using a pump-probe setup in sub-picosecond to nanosecond timescales. The fundamental laser pulses were generated by a Ti:Sapphire laser (Libra F, Coherent Inc., 800 nm, ~100 fs pulse width at repetition rate of 1 kHz). During the measurement, an excitation wavelength of 600 nm was used. The excitation fluence variation was carried out by inserting neutral density filters and

measuring the intensity of the laser beam after the filters. The white light was generated by passing through the fundamental laser pulse through a quartz cuvette filled with water. The transient absorption (TA) spectra at varying delay time were recorded by ExciPro TA spectrometer (CDP Inc.)[2].

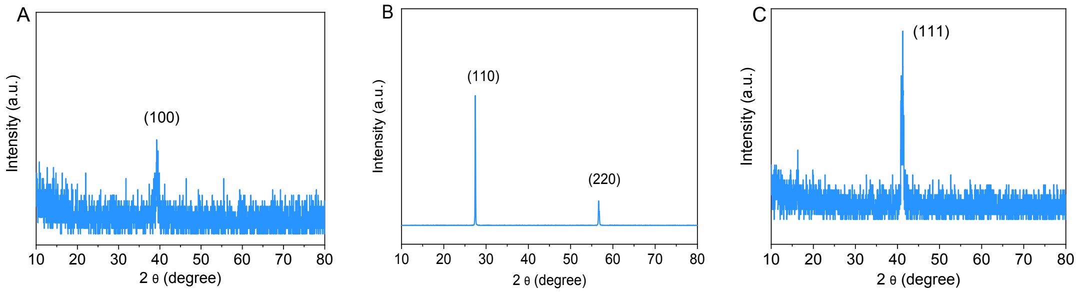

Figure S1. XRD patterns of the single crystal rutile TiO_2 substrates with three different facets of **A:** (100) **B:** (110) and **C:** (111).

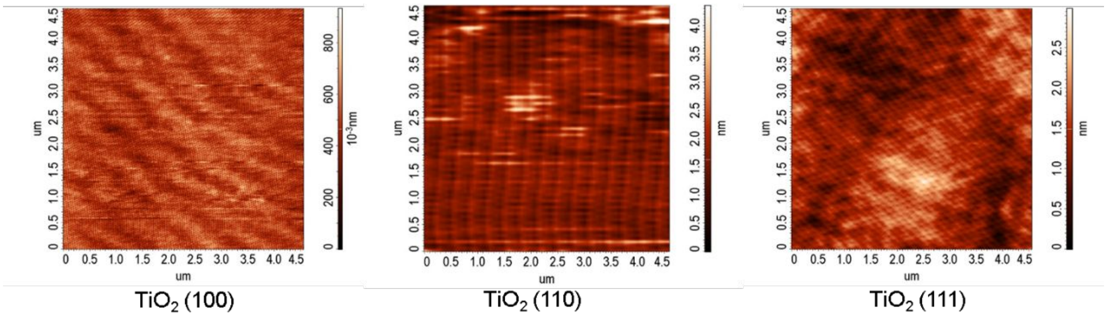

Figure S2. AFM images of the single crystal rutile TiO_2 substrates with three different facets. From these the average surface roughness were determined to be 0.1 nm, 0.3 nm, 0.3 nm for TiO_2 (100), TiO_2 (110) and TiO_2 (111) respectively.

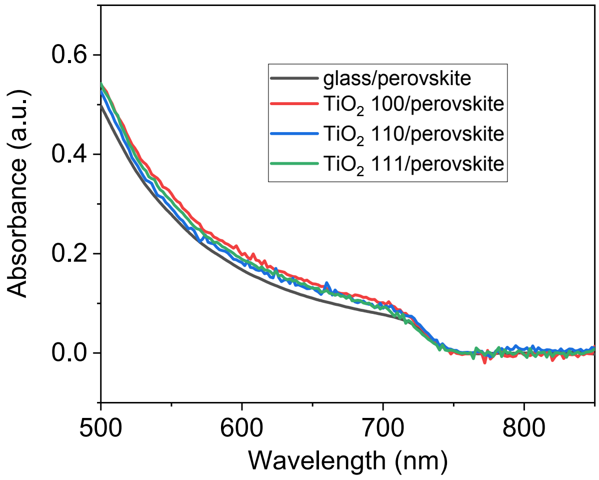

Figure S3. UV vis absorption of perovskite thin films deposited on different substrates.

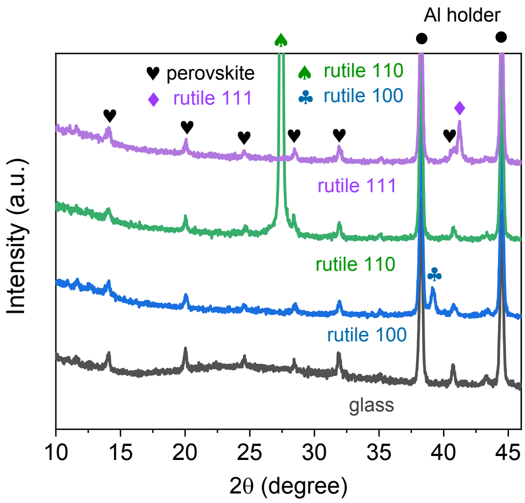

Figure S4. XRD patterns of a thin perovskite layer deposited on different substrates.

Table S1. Perovskite layers thickness and surface roughness on different substrates evaluated from ellipsometry result.

Substrate	Thickness (nm)	Surface roughness (nm)
glass	111	3.5
TiO_2 (100)	86	2
TiO_2 (110)	87	2
TiO_2 (111)	89	2

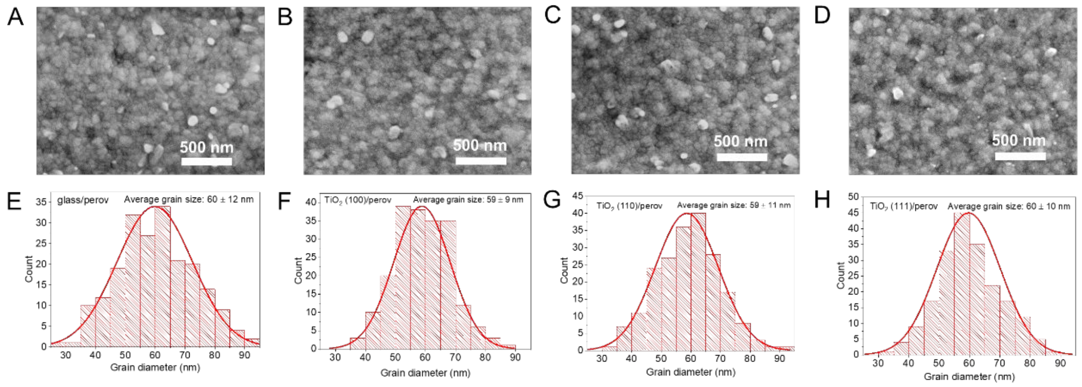

Figure S5. Top-down SEM images of perovskite layers on glass (A), TiO_2 (100) (B), TiO_2 (110) (C) and TiO_2 (111) (D) substrates. Determined grain size distribution of the perovskite layers on glass (E) and TiO_2 (100) (F), TiO_2 (110) (G) and TiO_2 (111) (H) substrates.

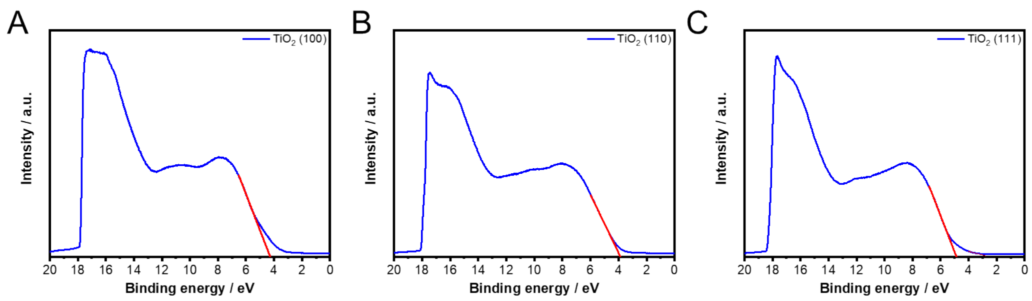

Figure S6. UPS results of single crystal TiO_2 (100) (A), TiO_2 (110) (B), TiO_2 (111) (C).

Table S2. Valence and conduction band position of single crystal TiO_2 determined from UPS measurements.

Crystal orientation	E_{sec} / eV	E_{VB} / eV	E_{VB} vs. vacuum / eV	E_{CB} vs. vacuum / eV
(100)	17.81	4.37	−7.78	−4.78
(110)	18.04	3.97	−7.15	−4.15
(111)	18.36	4.94	−7.80	−4.80

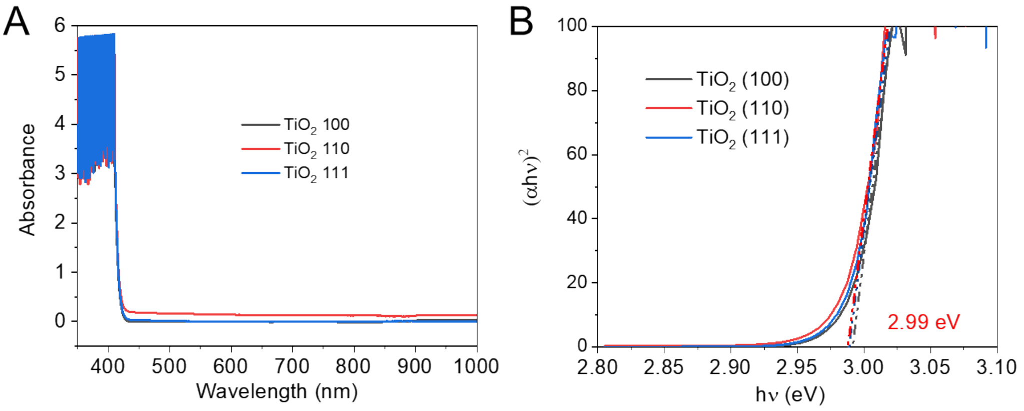

Figure S7. UV vis spectra of the single crystal rutile TiO_2 substrates with three different facets (A) and determined Tauc plots for a direct transition (B) of single crystals TiO_2.

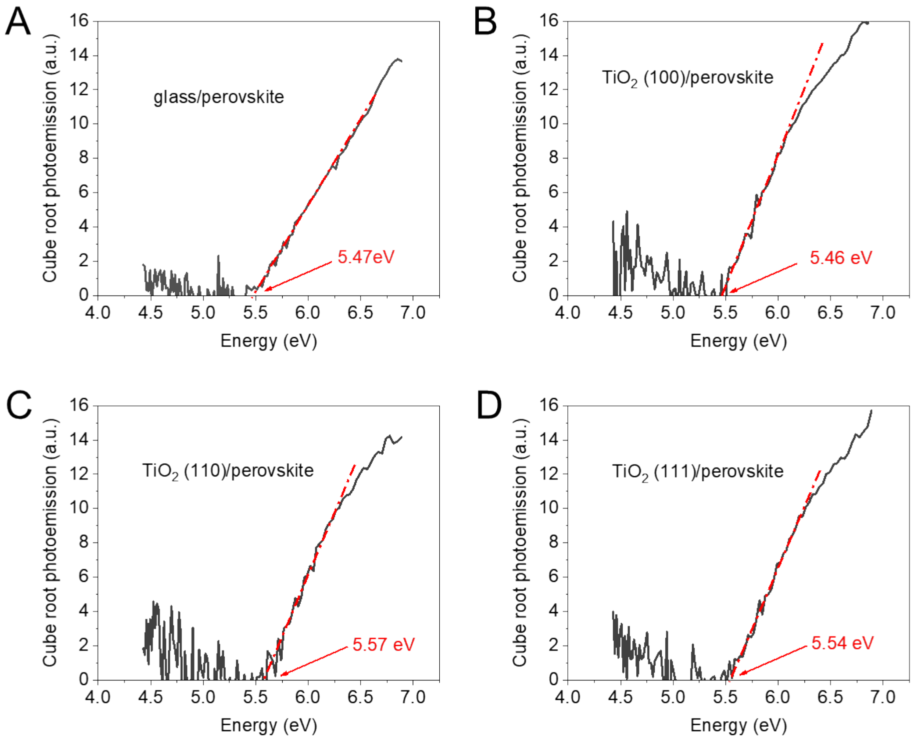

Figure S8. APS results of perovskite layers on glass (A), TiO_2 (100) (B), TiO_2 (110) (C), TiO_2 (111) (D) substrates.

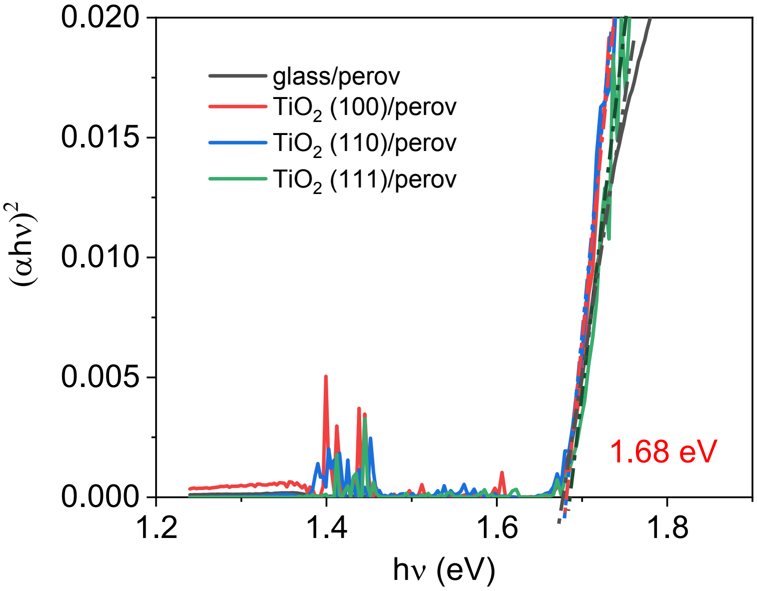

Figure S9. Determined Tauc plots of perovskite layers on different substrates derived for a direct transition from the UV-vis absorption spectra in **Figure S3**.

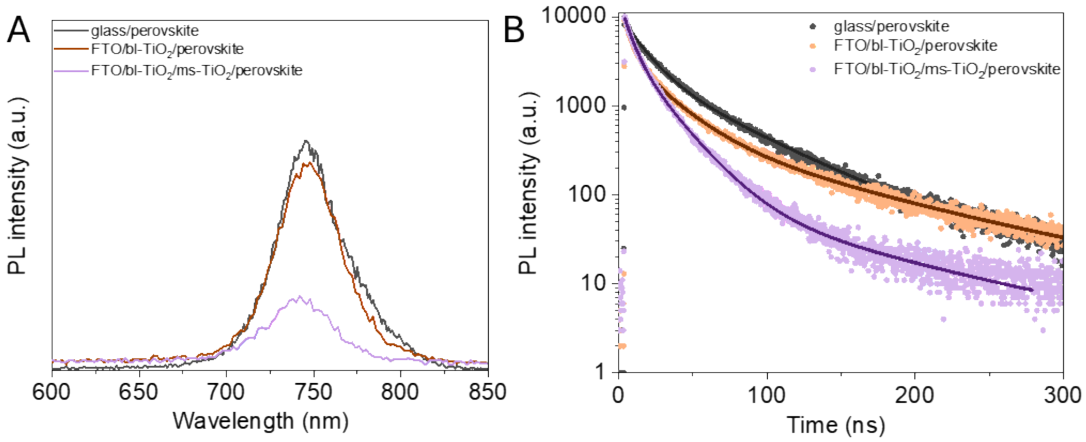

Figure S10. Steady state PL (A) and TRPL decay curve recorded at 750 nm (B) of perovskite on different substrates used as electron transfer layers in perovskite solar cells. Excitation wavelength: 467 nm. Excitation fluence: 29 nJ /cm^2.

Table S3. Photoluminescence quenching of perovskite on different substrates compared to glass substrate.

Sample	PL Intensity compared to glass/perovskite
Glass/perovskite	100%
TiO_2 (100)/perovskite	29.4%
TiO_2 (110)/perovskite	44.3%

TiO_2 (111)/perovskite	33.1%
FTO/bl-TiO_2/perovskite	92.7%
FTO/bl-TiO_2/mp-TiO_2/perovskite	37.3 %

Table S4. Fitting parameters and average lifetime of perovskite layer on different substrates.

	T_1 (ns)	A_1	T_2 (ns)	A_2	T_3 (ns)	A_3	T_{ave}
Glass/perovskite	6.9	0.43	29.9	0.51	97.2	0.06	24.0
TiO_2 (100)/perovskite	4.8	0.78	19.6	0.21	97.2	0.01	8.8
TiO_2 (110)/perovskite	5.5	0.55	23.9	0.41	97.2	0.04	16.7
TiO_2 (111)/perovskite	3.7	0.63	17.2	0.35	97.2	0.02	10.3
FTO/bl-TiO_2/perovskite	5.5	0.62	24.2	0.33	97.2	0.06	16.8
FTO/bl-TiO_2/mp-TiO_2/perovskite	6.3	0.60	20.5	0.38	97.2	0.01	12.8

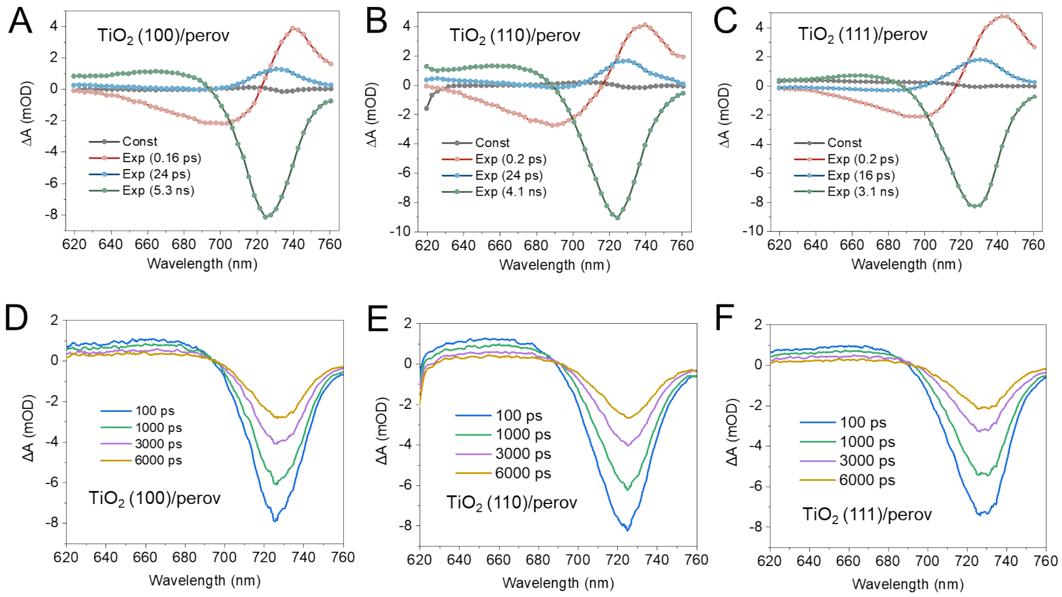

Figure S11. Decay associated spectra (DAS) of TiO_2 (100)/perovskite sample (A), TiO_2 (110)/perovskite sample (B), and TiO_2 (111)/perovskite sample (C). Transient absorption spectra (TAS) at various time delays of TiO_2 (100)/perovskite sample (D), TiO_2 (110)/perovskite sample (E), and TiO_2 (111)/perovskite sample (F). Excitation wavelength: 600 nm, excitation fluence: 5.7 μJ/cm^2.

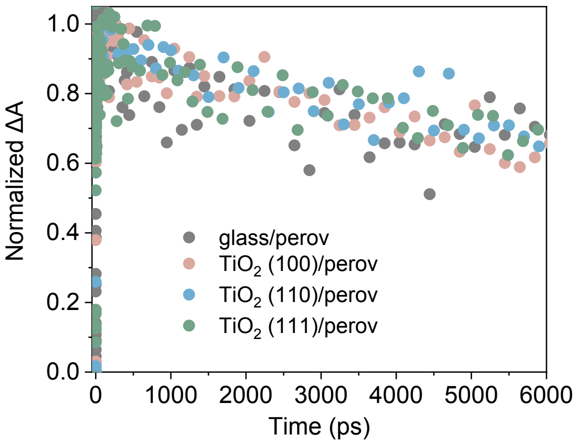

Figure S12. Transient absorption (TA) decay traces at 725 nm for perovskite on different substrates excited by a 600 nm wavelength laser at the fluence of 0.7 μJ/cm^2.

Table S5. Fitting parameters of TAS decay curves.

Sample	τ (ns)	β
Glass/perov	6.1 ± 0.1	0.64 ± 0.02
TiO_2 (100)/perov	5.3 ± 0.1	0.70 ± 0.01
TiO_2 (110)/perov	4.1 ± 0.1	0.64 ± 0.1
TiO_2 (111)/perov	3.1 ± 0.1	0.64 ± 0.2

Hot carrier fitting details:

The hot electron temperature (T_c) can be obtained by fitting the high energy tail of the band edge region of the GB signal in the TA spectra Using the Maxwell–Boltzmann distribution function:

$$\Delta A(E) = A_0 e^{-(E - E_f)/k_B T_c}$$

, where E_f is the quasi-Fermi level energy, and k_B is the Boltzmann constant. Note that the 600 nm excitation has a photon energy of 2.06 eV, while the perovskite in this study has a bandgap of 1.68 eV (according to 750 nm band edge from **Figure S9**), the excess energy is 0.38 eV, which should give a Kelvin temperature of 4410 K. According to our calculation, the hot carriers already reach ~1000 K after 10 fs, which means most of the energy were lost in the first 10 fs.

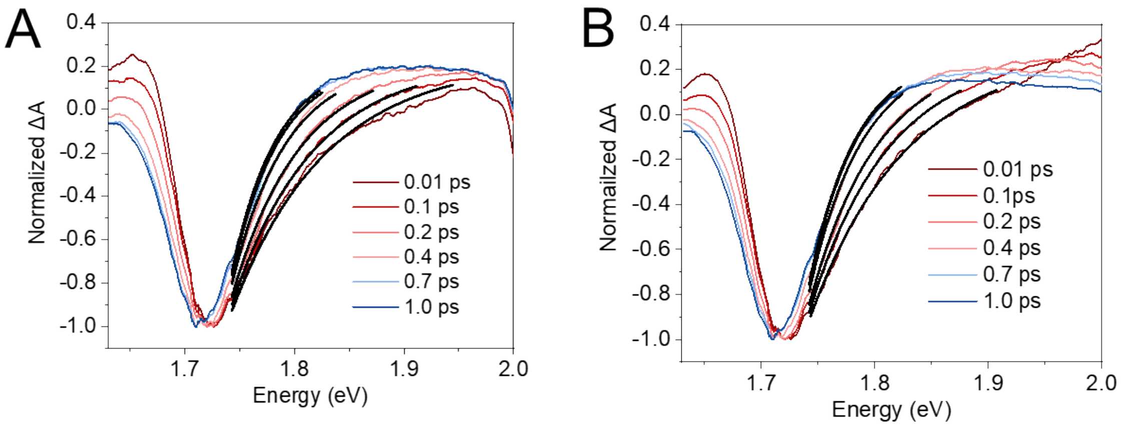

Figure S13. Hot carrier cooling fitting for TiO_2 (110)/perovskite (A) and TiO_2

(111)/perovskite (B) at excitation fluence of 5.7 μJ/cm^2.

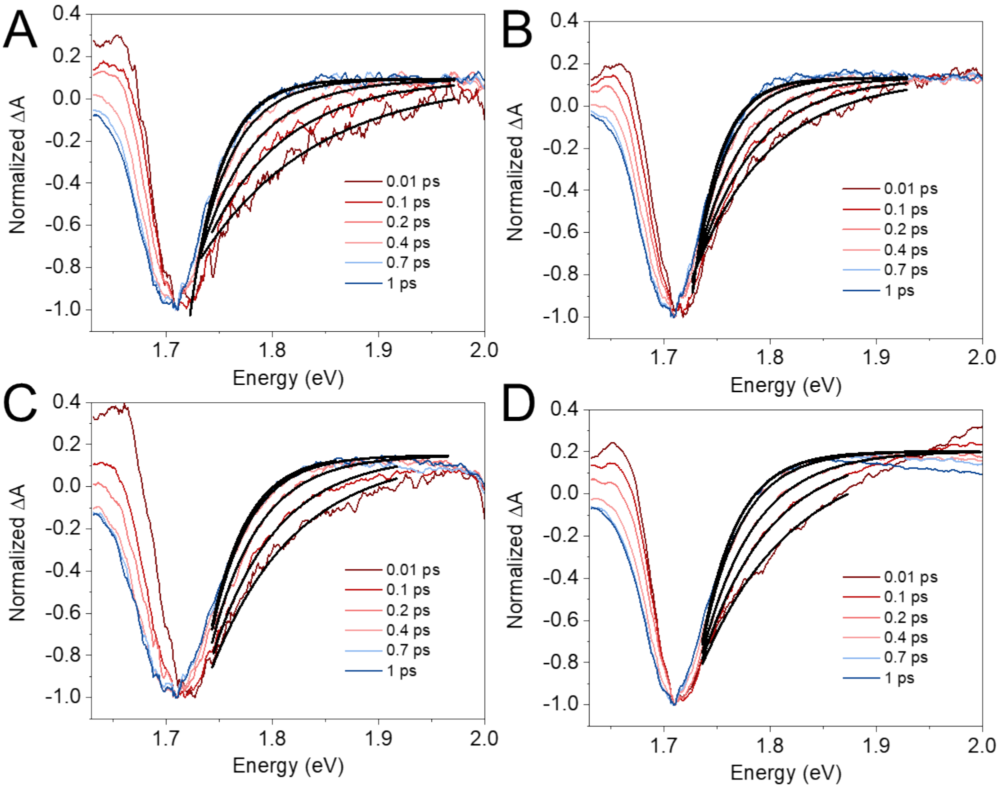

Figure S14. Hot carrier cooling fitting for glass/perovskite (A), TiO_2 (100)/perovskite (B), TiO_2 (110)/perovskite (C), TiO_2 (111)/perovskite (D) at excitation fluence of 2.8 μJ/cm^2.

References

(1) Baikie, I. D., Grain, A. C., Sutherland, J. & Law, J. Dual Mode Kelvin Probe: Featuring Ambient Pressure Photoemission Spectroscopy and Contact Potential Difference. *Energy Procedia* **2014**, *60*, 48–56.

(2) Virkki, K., Demir, S., Lemmetyinen, H. & Tkachenko, N. V. Photoinduced Electron Transfer in CdSe/ZnS Quantum Dot–Fullerene Hybrids. *J. Phys. Chem. C* **2015**, *119*, 17561–17572.
